# Thyroid Disorders Change the Pattern of Response of Angiotensinase Activities in the Hypothalamus-Pituitary-Adrenal Axis of Male Rats

**DOI:** 10.3389/fendo.2018.00731

**Published:** 2018-11-30

**Authors:** Ana B. Segarra, Isabel Prieto, Magdalena Martínez-Cañamero, Marc de Gasparo, Juan de Dios Luna, Manuel Ramírez-Sánchez

**Affiliations:** ^1^Department of Health Sciences, University of Jaén, Jaén, Spain; ^2^Cardiovascular and Metabolic Syndrome Adviser, Rossemaison, Switzerland; ^3^Department of Biostatistics, Medical School, University of Granada, Granada, Spain

**Keywords:** aminopeptidases, angiotensinases, euthyroid, hypothyroid, hyperthyroid, renin-angiotensin system

## Abstract

Thyroid disorders affect the hypothalamic-pituitary-adrenal axis with important consequences on the cardiovascular function in which the renin-angiotensin system plays a major role. Hypo and hyperthyroidism influence the classic main components of the renin-angiotensin system. However, the behavior of other elements of the renin-angiotensin system such as Ang III, Ang 2-10, Ang IV, or AT_4_, regulated by angiotensinase enzymes such as alanyl- (AlaAP), cystinyl- (CysAP), glutamyl- (GluAP), or aspartyl-aminopeptidase (AspAP), has not yet been described. In order to obtain a comprehensive view on the response of the renin-angiotensin system in the hypothalamic-pituitary-adrenal axis of animals with thyroid disorders, these enzyme activities were simultaneously analyzed fluorometrically, using arylamide derivatives as substrates in hypothalamus, anterior and posterior pituitary, adrenals and plasma of euthyroid, hypothyroid, and hyperthyroid rats, and their *intra*- and *inter*-tissue correlations were evaluated. The response is depending on the type of enzyme studied, its location and the thyroid status. Anterior pituitary, adrenals and plasma were mainly affected by the thyroid disorders. In the anterior pituitary, GluAP and AspAP increased in hypothyroid rats. In adrenals, AlaAP and CysAP decreased in hypothyroid whereas GluAP and AspAP decreased in hyperthyroid rats. In plasma, while AlaAP increased in hypo- and hyperthyroid rats, CysAP and GluAP decreased only in hyperthyroid. In comparison with euthyroid, *intra*-tissue correlations decreased in hypothyroid but *inter*-tissue correlations decreased mainly in hyperthyroid rats. Thyroid disorders also produced a disruption in the pattern of *inter*-tissue correlations observed in euthyroid. These results suggest that thyroid hormone levels hit components of the renin-angiotensin system and may influence the paracrine and endocrine cross talk between cells.

## Introduction

Thyroid disorders affect the hypothalamic-pituitary-adrenal axis (1–3) and have important consequences on the cardiovascular function in which the renin-angiotensin system plays a major role (4). Hypo and hyperthyroidism have demonstrated their influence on the classic main components of the renin-angiotensin system such as renin, angiotensin-converting enzyme, Ang II and its receptors AT_1_ and AT_2_ (4). The influence of the thyroid hormones on these components is heterogeneous and the final functional consequences of the thyroid disorders will therefore be the combined result of this diversity.

**Figure 1:**
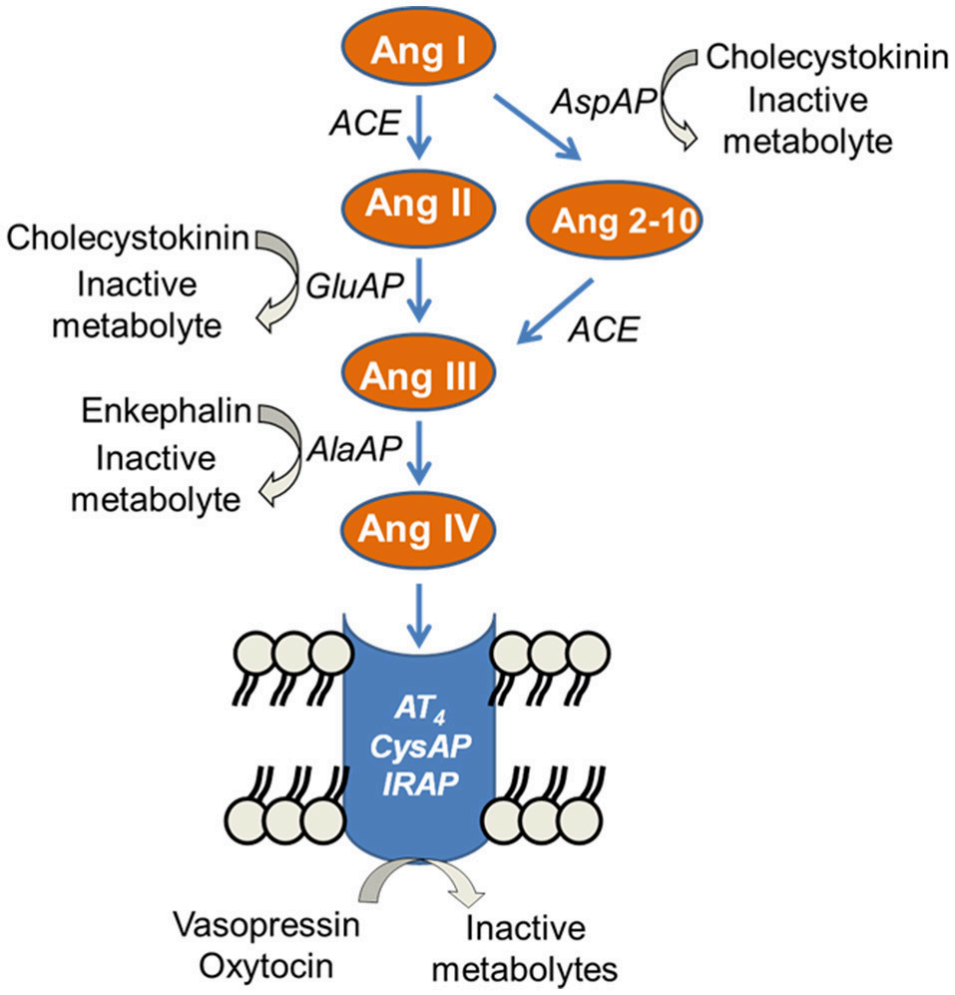
Partial representation of the enzymatic cascade of the renin-angiotensin system showing the steps in which the angiotensinases alanyl aminopeptidase (AlaAP), cystinyl aminopeptidase (CysAP), glutamyl aminopeptidase (GluAP), and aspartyl aminopeptidase (AspAP) are involved. Other substrates for the analyzed enzymes are indicated. (From 6 to 9).

The functional status of the endogenous peptidergic substrates is reflecting the proteolytic activity of the enzyme offering an accurate perspective for the possible functional changes of those enzymes under selective experimental conditions (5). We studied the activity of several aminopeptidases, called angiotensinases, in the hypothalamic-pituitary-adrenal axis as a reflection of the functional status of certain angiotensin peptides involved in blood pressure control. In the last decade, the renin-angiotensin system has experienced an increasing complexity with the discovery of new peptides with functions that opposed, in some cases, the classic actions exerted by Ang II. This is the case for other important active peptides of the renin-angiotensin system such as Ang 2-10, Ang III, or Ang IV whose generation and inactivation depend on the action of several angiotensinase activities such as glutamyl aminopeptidase (GluAP), responsible for the metabolism of Ang II to Ang III, aspartyl aminopeptidase (AspAP), responsible of the hydrolysis of Ang I to produce Ang 2-10, alanyl aminopeptidase (AlaAP) which metabolyzes Ang III to Ang IV and cystinyl aminopeptidase (CysAP), identified as vasopressinase or oxytocinase but also as the AT_4_ receptor and reported to be identical to the insulin-regulated aminopeptidase (6). The binding of Ang IV to its receptor (AT_4_, insulin-regulated aminopeptidase, vasopressinase, oxytocinase, CysAP) leads to the inhibition of its enzymatic activity, which reduces the metabolism of vasopressin or oxytocin (7). However, it should be taken into account that these enzymatic activities may also reflect the hydrolysis of other peptides such as cholecystokinin by AspAP and GluAP (8) or enkephalins by AlaAP (9) (Figure 1). Therefore, the activity of these enzymes reflects the functional status of their respective endogenous substrates as well as of the derived peptides (5).

All the components of the renin-angiotensin system have been recognized in all the tissues that integrate the hypothalamic-pituitary-adrenal axis where they act as local systems in coordination or not with the classic circulatory renin-angiotensin system (6). In order to obtain a more comprehensive view of the response of the renin-angiotensin system in the hypothalamus-pituitary-adrenal axis of animals with thyroid disorders, the pattern of behavior of these activities correlating *intra*-tissue and *inter*-tissues was analyzed fluorometrically in hypothalamus, anterior- and posterior-pituitary, adrenal gland, and plasma of euthyroid, hypothyroid, and hyperthyroid rats, using arylamide derivatives as substrates. For this enlarged study we also used some previously published data framed for other purposes (10–12). Prieto et al. (10) described the activity of AlaAP, GluAP, and AspAP in hypothalamus and pituitary of rats with thyroid disorders. Wangensteen et al. (11) reported a divergence between the high renal expression of GluAP and the low level of its activity determined in kidney and plasma of hyperthyroid rats. Finally, Segarra et al. (12) reported a coordinated modification of CysAP (insulin-regulated aminopeptidase) and pGluAP (TRH-degrading activity) between hypothalamus, liver and plasma, possibly linked to changes in liver metabolism depending on the thyroid status of rats.

## Materials and methods

### Animals and experimental procedures

The experimental procedures for animal use and care were in accordance with European Communities Council Directive 86/609/EEC and was approved by the bioethics committee of the University of Jaén. Twenty male Sprague-Dawley rats (Charles River Laboratories, Barcelona, Spain) weighing 180–200 g were used in this study. The animals were randomly divided into three groups to further develop euthyroid (*n* = 7), hypothyroid (*n* = 7), and hyperthyroid (*n* = 6) rats. They were housed in standard laboratory cages and kept in a temperature-controlled room (23–25°C) with a 12 h/12 h light/dark schedule. Laboratory food and water were provided *ad libitum*. Hyperthyroidism was induced with daily subcutaneous injections of tetraiodothyronine (T4) (Sigma-Aldrich Co., St. Louis, MO, 300 μg/kg/day) for 6 weeks. Hypothyroid rats were developed with 0.03% methimazole (Sigma) in the drinking water for 6 weeks (13). The group of euthyroid rats was treated with subcutaneous administration of the same solution, in the same conditions as the hyperthyroid group but without T4. After 6 weeks of treatment, animals were anesthetized with equithensin (2 ml per kg body weight) (equithensin contained 42.5 g/l chloralhydrate dissolved in 19.76 ml ethanol, 0.396 l/l propylenglycol, 21.3 g/l magnesium sulfate, and 9.72 g/l Nembutal in distilled water) injected intraperitoneally. Blood samples were obtained from the left cardiac ventricle and centrifuged for 10 min at 2,000 g to obtain plasma which was stored at −20°C. Rats were then perfused with saline solution through the left cardiac ventricle. The total brain, anterior pituitary, posterior pituitary, and adrenal (pooled left and right) were quickly removed (>60 s) and cooled in dry ice. The hypothalamus (pooled left and right) was dissected according to the stereotaxic Paxinos & Watson atlas (14). The selected area was between 7.7 and 3.7 mm anterior to the interaural line. From these samples, the membrane-bound fraction was obtained as previously described (15). Briefly, samples from tissues were homogenized in a hypoosmolar medium (10 mM HCl–Tris buffer, pH 7.4). The homogenates were ultracentrifuged at 100,000 g for 30 min at 4°C. To get the membrane-bound fraction, the pellets obtained in the above ultracentrifugation were homogenized (again) in HCl–Tris buffer (pH 7.4) containing 1% of detergent Triton X-100. After (new) ultracentrifugation (100,000 g, 30 min, 4°C), the obtained supernatants were used to determine enzymatic activities and proteins in triplicate. The adsorbent polymeric Bio-beads SM−2 (Sigma, 100 mg/ml) (shaking the samples for 2 h at 4°C) was used to remove the Triton detergent from the medium and fully recover the enzyme activity.

### Enzymatic determinations

The activities of glutamyl- (GluAP), aspartyl- (AspAP), alanyl- (AlaAP), and cystinyl-aminopeptidase (CysAP), were determined fluorometrically using arylamide derivatives, as substrates as previously described (16). Briefly, AlaAP and CysAP were measured using Ala- or Cys-ß-naphthylamide as substrates: 10 μl of each supernatant and plasma were incubated for 30 min at 25°C with 1 ml of the substrate solution: 2.14 mg/100 ml of Ala-ß-naphthylamide or 5.53 mg/100 ml of Cys-ß-naphthylamide. 10 mg/100 ml bovine serum albumin (BSA), and 10 mg/100 ml dithiothreitol in 50 mM of phosphate buffer, pH 7.4, for AlaAP and 50 mM HCl-Tris buffer, pH 6, for CysAP. AspAP was studied with Asp-ß-naphthylamide as substrate: 10 μl of each supernatant and plasma were incubated for 120 min at 37°C with 1 ml of the substrate solution (2.58 mg/100 ml Asp-ß-naphthylamide, 10 mg/100 ml bovine serum albumin, 10 mg/100 ml dithiothreitol, and 39.4 mg/100 ml MnCl2 in 50 mmol/l HCl-Tris buffer, pH 7.4). GluAP was evaluated using Glu-ß-naphthylamide as substrate: 10 μl of each supernatant was incubated during 120 min at 37°C with 1 ml of the substrate solution (2.72 mg/100 ml Glu-ß-naphthylamide, 10 mg/100 ml bovine serum albumin, 10 mg/100 ml dithiothreitol and 0.555 g/100 ml CaCl2 in 50 mmol/l HCl-Tris, pH 7.4). The enzymatic reaction was stopped by adding 1 ml of 0.1 mol/l of acetate buffer (pH 4.2). The quantity of ß-naphthylamine released as a result of enzymatic activity was determined fluorometrically at an emission wavelength of 412 nm with an excitation wavelength of 345 nm. Proteins were quantified in triplicate by the method of Bradford (17). Specific AlaAP, CysAP, GluAP, and AspAP activities were expressed as pmol of Ala-, Cys-, Glu-, or Asp-ß-naphthylamide hydrolyzed per min per mg of protein. The results of the fluorogenic assays were linear with respect to time of hydrolysis and protein content.

### Statistical analysis

A descriptive analysis was performed for each of the variables (enzymatic activities) in each group (euthyroid, hypothyroid, and hyperthyroid) and in each region. We used mean and standard errors for presenting the data.

In order to assess correlation between variables in the same region or between variables in different regions Pearson's correlation coefficient was used. Because a huge number of correlations were performed, these correlations were adjusted using Bonferroni's penalization for each correlation's matrix referenced before. Due to the essentially exploratory nature of this paper, a greater penalization was not used. Correlations with Bonferroni's significance levels lower than 0.05 were declared significant.

For each of the variables, an analysis of their variations among groups and regions was performed using a two way anova design: the first factor was a fixed effects factor (group) with euthyroid, hypothyroid and hyperthyroid categories; a second factor (rat) that was a random effect factor nested in group with 7, 7, and 6 levels, respectively. A third factor (region) that was a repeated measure factor with fixed effects, crossed with groups. In these models, effects of fixed factors were considered and the interaction group per region was assessed first with a Bonferroni's penalization for the four designs used (one for each variable). All interactions were significant at the cited level. If interaction was significant, pairwise comparisons were performed using Tukey's penalizations which was done because of the balance of the samples size. These comparisons were declared significant for a penalized Tukey's *P*-value. Computations were performed using STATA 14.1.

## Results

**Figure 2:**
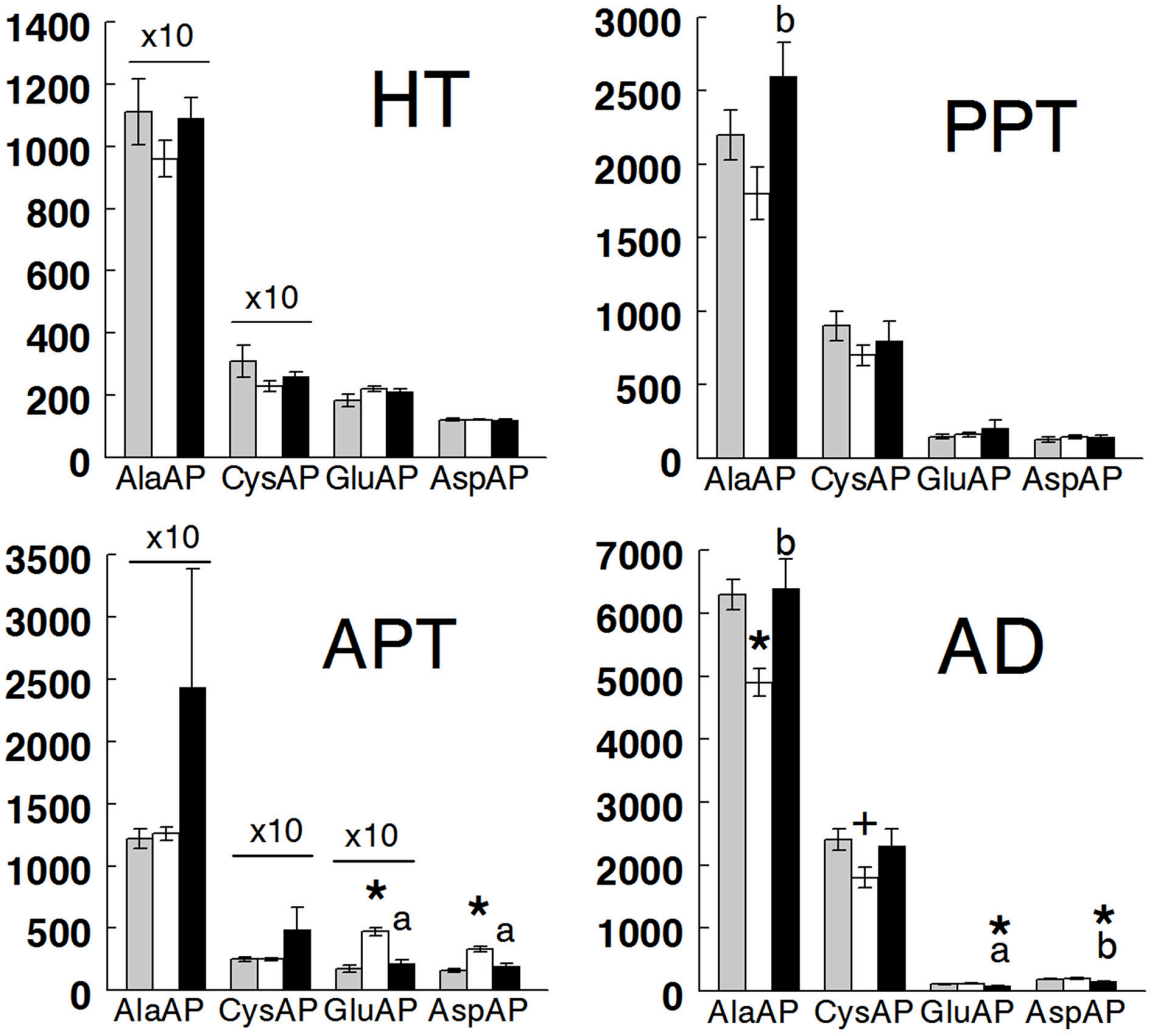
Mean ± SEM levels of alanyl aminopeptidase (AlaAP), cystinyl aminopeptidase (CysAP), glutamyl aminopeptidase (GluAP), and aspartyl aminopeptidase (AspAP) activities expressed as pmol/min/mg of proteins in the anterior hypothalamus (HT) anterior pituitary (APT), posterior pituitary (PPT), and adrenal gland (AD) of euthyroid (gray bars), hypothyroid (white bars) and hyperthyroid (black bars) rats. (*) indicates a significant difference of *p* < 0.001 vs. euthyroid rats. (+) indicates a significant difference of *p* < 0.05 vs. euthyroid rats. (a) indicates a significant difference of *p* < 0.001 vs. hypothyroid rats. (b) indicates a significant difference of *p* < 0.01 vs. hypothyroid rats. For clarity of the figure, the actual values of AlaAP and CysAP in HT and AlaAP, CysAP, and GluAP in APT have been adjusted by a coefficient factor 10.

**Figure 3:**
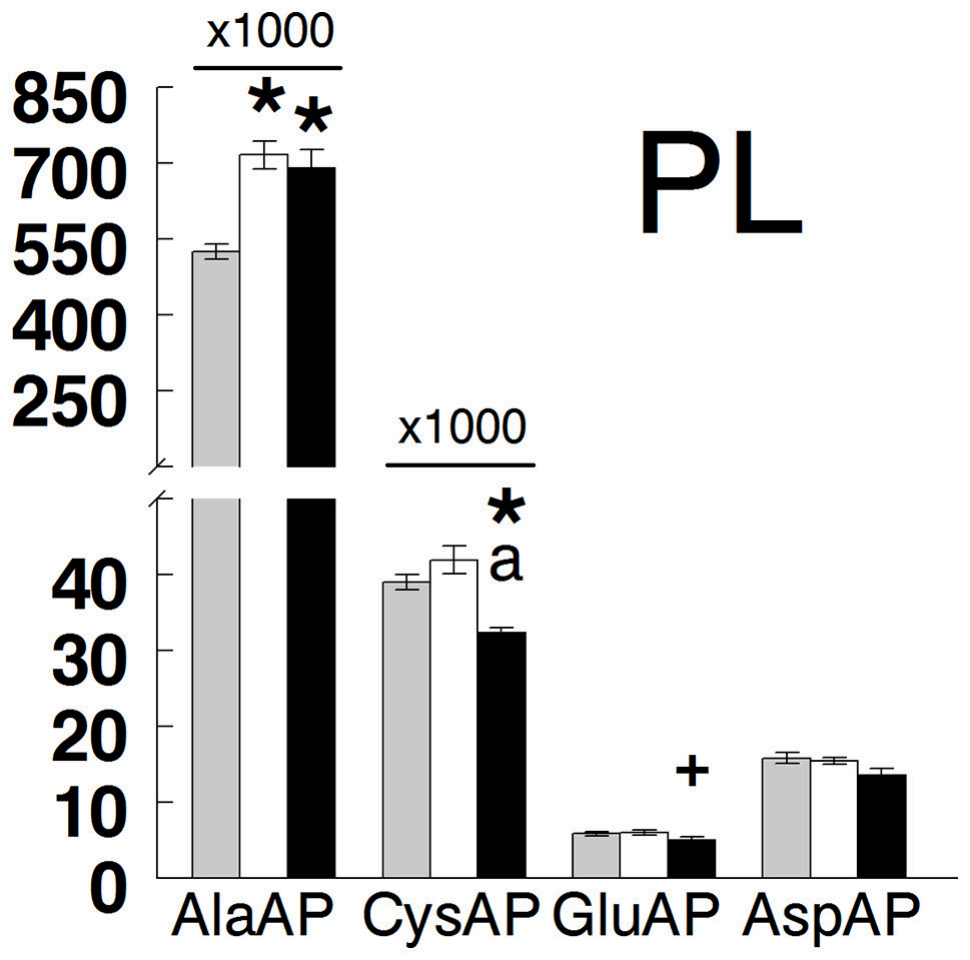
Mean ± SEM levels of alanyl aminopeptidase (AlaAP), cystinyl aminopeptidase (CysAP), glutamyl aminopeptidase (GluAP), and aspartyl aminopeptidase (AspAP) activities expressed as pmol/min/mg of proteins in the plasma (PL) of euthyroid (gray bars), hypothyroid (white bars) and hyperthyroid (black bars) rats. (*) indicates a significant difference of *p* < 0.001 vs. euthyroid rats. (+) indicates a significant difference of *p* < 0.05 vs. euthyroid rats. (a) indicates a significant difference of *p* < 0.001 vs. hypothyroid rats. For clarity, the actual values of AlaAP and CysAP have been multiplied by a coefficient factor 1000 in the figure.

Results are presented in Figure 2 (hypothalamus, anterior and posterior pituitary, and adrenals) and Figure 3 (plasma) and in Table 1. All tissues demonstrated the highest levels of activity for AlaAP followed by CysAP and the lowest for GluAP and AspAP in the three groups studied. In hypothalamus, there were no differences between groups in any of the enzymatic activities analyzed. In the anterior pituitary, no differences between groups were demonstrated for AlaAP and CysAP. However, GluAP and AspAP demonstrated significant higher levels in hypothyroid than in euthyroid and hyperthyroid rats (*p* < 0.001). In posterior pituitary, AlaAP was significantly higher in hyperthyroid than hypothyroid (*p* < 0.01) rats without difference with euthyroids. No differences between groups were demonstrated with the other enzyme activities. In the adrenals, AlaAP was significantly lower in hypothyroid than euthyroid (*p* < 0.001) and hyperthyroid (*p* < 0.01) rats. The lowest levels of CysAP were observed in hypothyroid rats, differing significantly only with euthyroids (*p* < 0.05). GluAP activity was lower in hyperthyroid than euthyroid (*p* < 0.001) and hypothyroid (*p* < 0.001) rats. AspAP was also lower in hyperthyroid than euthyroid (*p* < 0.001) and hypothyroid (*p* < 0.01) rats. In plasma, AlaAP was higher in hypothyroid and hyperthyroid rats than euthyroids (*p* < 0.001). In contrast, CysAP was lower in hyperthyroid than euthyroid and hypothyroid rats (*p* < 0.001) whereas GluAP was lower (*p* < 0.05) in hyperthyroid than euthyroid rats. No differences between groups were observed for AspAP.

**Table 1:** Intra-tissue and inter-tissue correlations between angiotensinase activities.

**Euthyroid**		**Hypothyroid**		**Hyperthyroid**	
**Correlation**	***r***	**Correlation**	***r***	**Correlation**	***r***
HT AlaAP vs. HT CysAP	+0.903	APT AlaAP vs. APT CysAP	+0.937	HT AlaAP vs. HT CysAP	+0.994
APT AlaAP vs. APT CysAP	+0.981	***PPT GluAP*** **vs*****. PL CysAP***	*−**0.876***	APT AlaAP vs. APT CysAP	+0.999
APT GluAP vs. APT AspAP	+0.942	AD CysAP vs. PL CysAP	+0.935	APT GluAP vs. APT AspAP	+0.942
AD CysAP vs. AD AspAP	+0.897	***AD AspAP*** **vs**. ***PL AspAP***	*−**0.921***	***APT AlaAP*** **vs*****. PPT AspAP***	*−**0.920***
HT CysAP vs. APT AlaAP	+0.894			APT AspAP vs. AD AspAP	+0.965
HT CysAP vs. APT CysAP	+0.883				
***HT GluAP vs. APT AlaAP***	*−**0.898***				
APT GluAP vs. PL AlaAP	+0.875				

*Significant intra- and inter-tissue correlations observed between AlaAP, alanyl aminopeptidase; CysAP, cystinyl aminopeptidase; GluAP, glutamyl aminopeptidase; AspAP, aspartyl aminopeptidase activities measured in HT, hypothalamus; APT, anterior pituitary; PPT, posterior pituitary; AD, adrenal gland; PL, plasma of euthyroid, hypothyroid, and hyperthyroid rats. Pearson's correlation coefficients (r) are indicated. Negative correlations are in red and italic. Gray background highlights the inter-tissue correlations*.

The comparison between regions demonstrated that AlaAP was significantly higher in plasma than in the other targets of all groups. CysAP was also higher in plasma than in the tissues in all groups but higher in the anterior pituitary than in the posterior one in hyperthyroid rats. GluAP was higher in the anterior pituitary than in hypothalamus, posterior pituitary, adrenals, and plasma in all groups. AspAP was lower in plasma than in the different tissues of all groups. AspAP was higher in adrenals than in hypothalamus in euthyroid and hypothyroid rats, higher than in posterior pituitary in euthyroids and lower than in the anterior pituitary in hypothyroid rats. Finally, AspAP was higher in the anterior pituitary than in hypothalamus of hypothyroid and hyperthyroid rats and in posterior pituitary of hypothyroid rats.

### Correlational analysis

**Figure 4:**
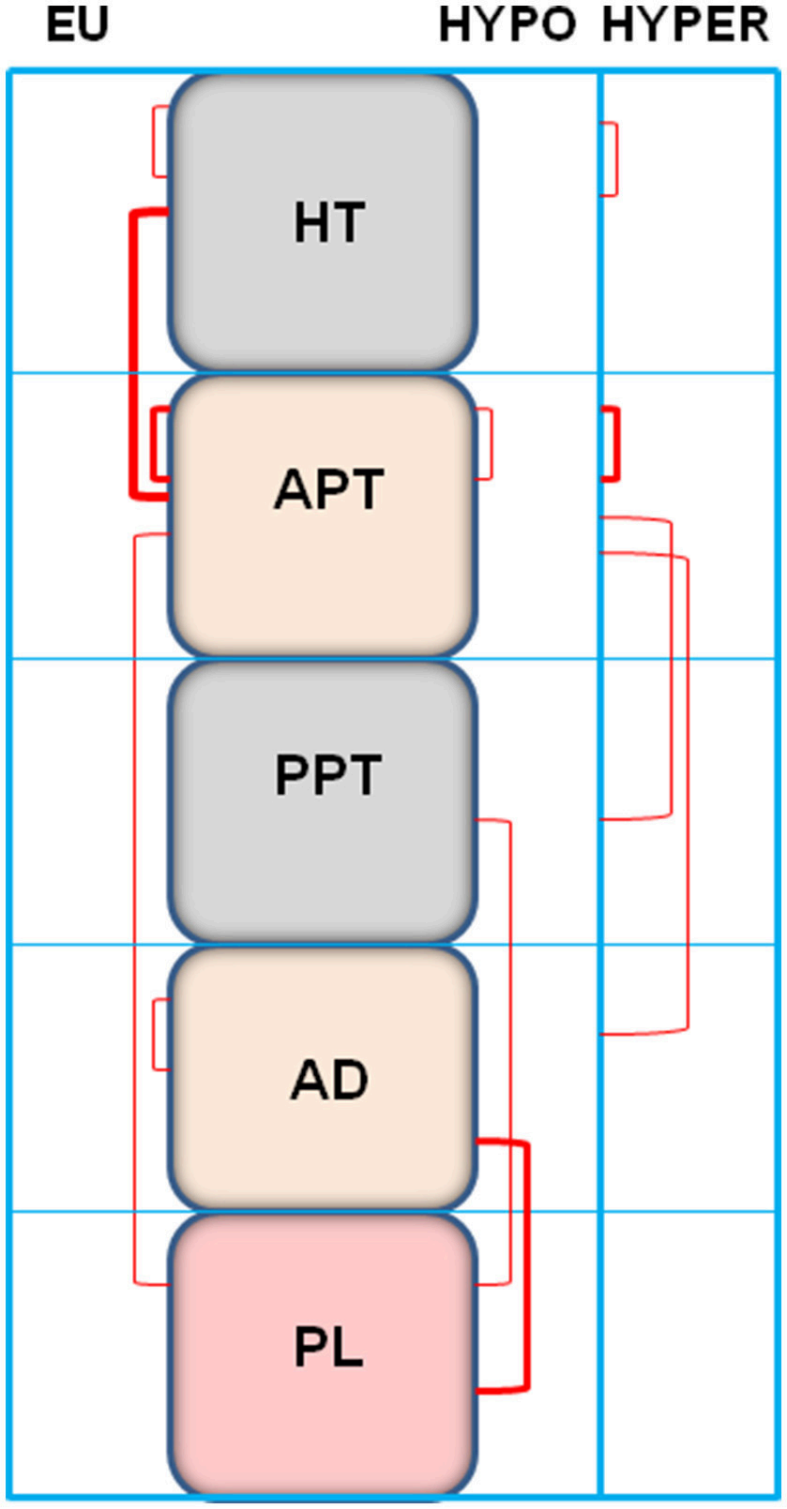
Representation of the significant *intra*- and *inter*-tissue correlations between angiotensinase activities (reported in Table 1) of the anterior hypothalamus (HT) anterior pituitary (APT), posterior pituitary (PPT), adrenal gland (AD), and plasma (PL) of euthyroid (EU), hypothyroid (HYPO), and hyperthyroid (HYPER) rats. The thickness of lines is proportional to the number of correlations observed.

The values of the correlational study are presented in Table 1 and represented in Figure 4. The correlations pattern changes significantly depending on the analyzed group. Considering the *intra*-tissue and *inter*-tissue correlations, the most part of *inter*-tissue correlations were observed in the euthyroid group. These correlations were reduced in the hypothyroid and in the hyperthyroid group. The euthyroid group showed the higher number of *intra*-tissue correlations that were slightly reduced in hyperthyroid but mainly in hypothyroid rats (Table 1). The type of *inter*-tissue correlations also depends on the thyroid status. Whereas, the most part of the correlations in the euthyroid group were observed between hypothalamus and anterior pituitary, they were displaced in hypothyroids to correlations between posterior pituitary and adrenals vs. plasma. In the hyperthyroid group, the anterior pituitary correlates negatively with the posterior pituitary and positively with adrenals (Table 1).

## Discussion

The response to the increased or decreased thyroid hormones in the tissues studied was clearly heterogeneous and the measured levels of angiotensinase activities depended on the type of enzyme, the location and the thyroid status. Therefore, the diverse direct or indirect action of thyroid hormones on the components of the renin-angiotensin system may condition the final response of the system. The behavior of the measured enzymatic activities gives us an idea of the complex relations that can be established between the various components of the system.

Table 2 summarizes the published response of the hypothalamus-pituitary-adrenal axis to hypo- and hyperthyroidism (1–4, 12, 18) and includes the possible consequences of the present findings. An activation of the hypothalamus-pituitary-adrenal axis in hyperthyroidism and an inhibition of the axis in hypothyroidism are generally accepted (1–4). A reduction of corticosterone (2) together with a reduction in the adrenal weight (3) was described in hypothyroid rats but the contrary was observed in hyperthyroid (1). In addition, a decrease of plasma angiotensin-converting enzyme and Ang II in hypothyroid and an increase of these renin-angiotensin system components in hyperthyroid rats was reported (4) (Table 2). Clearly, the renin-angiotensin system plays a major role in the paracrine and endocrine regulation of hormone release in the hypothalamus, anterior pituitary (19) and adrenals (20).

**Table 2:** Response of the hypothalamus-pituitary-adrenal axis to the hypo- and hyperthyroidism in comparison with euthyroid status.

**Tissue**	**Hypothyroid**	**Hyperthyroid**
Hypothalamus	↓CRH mRNA (2) ↓AVP (18) ↓OX (18)	=AVP (18) = OX (18)
Anterior Pituitary	↑GluAP (↑***Ang III***, ↓***Ang II***) ↑AspAP (↑***Ang 2-10***, ↓***Ang I***)	
Posterior Pituitary	=AVP (18) = OX (18)	=AVP (18) ↓OX (18)
Adrenal	↓Corticosterone (2) ↓Adrenal weight (3) ↓AlaAP (↑***Ang III***, ↓***Ang IV*****)** ↓CysAP (*↑**AVP*****)**	↑Corticosterone (1) ↑Adrenal weight (1) ↓GluAP (↑***Ang II***, ↓***Ang III*****)** ↓AspAP (↑***Ang I***, ↓***Ang 2-10*****)**
Plasma	=AVP (18) ↓ACE (4) ↓Ang II (4) ↑OX (18) ↑AlaAP (↑***Ang IV***, ↓***Ang III*****)**	=AVP (18) ↑ACE (4) ↑Ang II (4) ↑OX (18) ↑AlaAP (↑***Ang IV***, ↓***Ang III*****)** ↓CysAP (*↑**AVP*****)** (12)

*In parenthesis, the reference number from which this information was obtained. In italics and bold are the suggested consequences of the enzymatic variations observed in the present study and from Segarra et al. (12). ACE, angiotensin-converting enzyme; AVP, arginine vasopressin; CRH, corticotropin releasing hormone; OX, oxytocin. (From 1 to 4, 12, 18)*.

In the present study, the main changes were observed for GluAP and AspAP in anterior pituitary, for all enzymatic activities studied in adrenals and for AlaAP and CysAP in plasma. The role of the renin-angiotensin system in the endocrine release of hormones from anterior pituitary and adrenals may therefore be substantially altered in hypo and hyperthyroid rats. In the anterior pituitary there was a clear increase of GluAP and AspAP in hypothyroids without change in AlaAP and CysAP. According to the enzymatic renin-angiotensin system cascade (Figure 1), these results suggest an increased formation of Ang III and Ang 2-10 in hypothyroid rats.

In adrenals, our data demonstrated a significant decrease of AlaAP and CysAP in hypothyroid rats. These results of AlaAP may indicate a longer action of Ang III due to the reduction of its metabolism which could be linked to a greater release of aldosterone from the adrenal gland (21). In addition, the reduction of CysAP may indicate a longer action of arginine-vasopressin in the adrenals of hypothyroids. These results are in agreement with those reported by Yeum et al. (22) demonstrating an increase of aquaporins in hypothyroid rat kidney. Taken together, these results may explain in part the decreased diuresis observed in hypothyroidism (23, 24). In the adrenals of hyperthyroid rats, our data suggest a longer action of Ang I and Ang II due to the decreased activity of GluAP and AspAP. In plasma, there was an increase of AlaAP in hypothyroids which suggests a higher formation of Ang IV than in euthyroid rats. There was also a higher plasma AlaAP activity in hyperthyroids accompanied by a decrease in CysAP. According to the reported effect of Ang IV on AT_4_ which reduces insulin-regulated aminopeptidase (CysAP) activity (7), these results suggest a higher formation of Ang IV which fits with the lower levels of CysAP and suggest a longer action of arginine-vasopressin in hyperthyroid subjects. These results are also in agreement with Mogulkoc and Baltaci (25) who reported increased plasma levels of arginine-vasopressin in rats with induced experimental hyperthyroidism.

**Figure 5:**
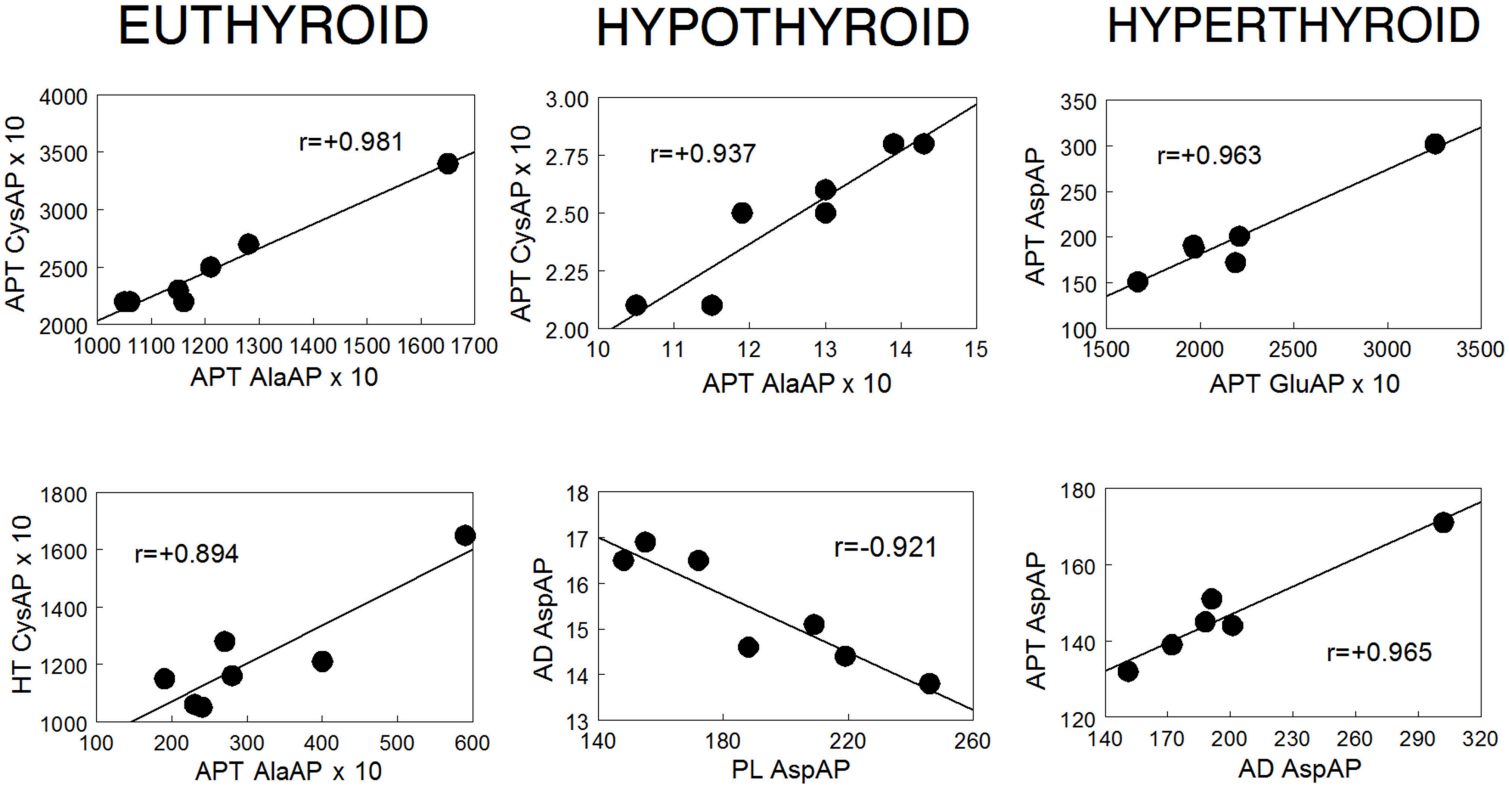
Examples of significant *intra*-tissue and *inter*-tissue correlations observed in euthyroid, hypothyroid and hyperthyroid rats.

In order to have an overview of the behavior of angiotensinases in the hypothalamus-pituitary-adrenal axis depending on thyroid status, we carried out a correlational study among the different tissues analyzed, in each of the groups studied. The results (Figures 4, 5 and Table 1) demonstrated clear differences between the groups. In euthyroid rats there were correlations essentially between hypothalamus and anterior pituitary and between the anterior pituitary and plasma. However, this pattern changed considerably with thyroid disorders. In hypothyroid rats, the absence of thyroid hormones led to (1) the loss of *inter*-tissue correlations involving hypothalamus and anterior pituitary, (2) the emergence of other correlations not existing in the euthyroid group such as between posterior pituitary, adrenals and plasma, and (3) a decrease of *intra*-tissue correlations to only one in the anterior pituitary. However, the increase of thyroid hormones allows the recovery of *intra*-tissue correlations that involve hypothalamus and anterior pituitary but to a lesser degree than those correlations observed in the euthyroid rats. Hyperthyroidism also changed the pattern of the *inter*-tissue correlations observed in euthyroid rats showing significant ones between anterior pituitary vs. posterior pituitary and adrenals.

In relation with the above-mentioned possible influence of some enzyme activities on water balance control, we previously reported significant correlations between AlaAP and CysAP (vasopressinase) activities of the posterior pituitary and of the adrenals in a rat saline model of volume hypertension (26) in which a marked water retention was produced. These results suggested a relationship between these activities, their locations and the aqueous control. The present observed correlations between anterior and posterior pituitary, adrenals and plasma in hypothyroid and hyperthyroid rats, involving vasopressinase and other enzymatic activities, might be related to the alterations in water retention previously described in these disorders (23, 25).

The present observations suggest an influence of thyroid hormones in the stimulation or inhibition of *paracrine* and *endocrine* communications, in which the angiotensinase activities (and consequently their endogenous substrates) are implicated. The results suggested a reduction of the *paracrine* communication in hypothyroid rats and also a slight reduction of the endocrine one in hyperthyroid rats (Table 1, Figure 4). We could therefore hypothesize that the level of aminopeptidase activities and their correlations may be due to the influence of thyroid hormones on the gene expression of the selected enzymes. However, in addition to this classic mechanism of action exerted by thyroid hormones, other factors may be involved as enzyme gene expression do not necessarily parallel with enzyme activity. Post-translational factors may also be involved in these responses (11). A direct *in vitro* influence of cholesterol and sex steroids on aminopeptidase activities was reported [reviewed in (27)]. It is known that thyroid disorders change the plasma levels of cholesterol and sex steroids. These changes together with a possible complementary direct influence of thyroid hormones (11) may also differentially affect the aminopeptidases of the tissues of the hypothalamus-pituitary-adrenal axis resulting in diverse patterns of correlations. The local and systemic environment created in hypo- and hyperthyroidism may differentially influence paracrine and endocrine signals leading to a different pattern of correlations in hypothyroid and hyperthyroid rats. A similar mechanism connecting adipocyte dysfunction with changes in paracrine and endocrine signals has been proposed to promote cancer development and its progression through alterations in the microenvironment (28).

Although we have not measured the endogenous substrates of the analyzed enzymes, which constitutes a limitation of our study, the marked changes observed in the pattern of correlations depending on the thyroid status represents an important observation: they may reflect alterations in the mechanism underlying the paracrine and/or endocrine communication between tissues of the hypothalamus-pituitary-adrenal axis. Therefore, the mechanism by which this process is carried out and the consequences of these changes remains to be elucidated. The possibility that the levels of thyroid hormones determine the degree of intercellular communication involving the endogenous substrates of the enzymes studied in the present work is of a special interest due to the possibility of controlling such enzymes through the use of specific activators or inhibitors (6).

In conclusion, the response of angiotensinase activities in the hypothalamus-pituitary-adrenal axis depends on the type of enzyme involved, its location and the thyroid status. Anterior pituitary, adrenals, and plasma were mainly affected. In the anterior pituitary, GluAP and AspAP increased in hypothyroid rats. In adrenals, AlaAP and CysAP decreased in hypothyroid and GluAP and AspAP decreased in hyperthyroids. In plasma, while AlaAP increased in hypothyroid and hyperthyroid rats, CysAP decreased only in hyperthyroids. In addition, in comparison with euthyroids, *intra*-tissue correlations decreased in hypothyroids and *inter*-tissue correlations decreased in both thyroid disorders but mainly in hyperthyroid rats. These results suggest that thyroid hormones influence the intercellular communications in which the renin-angiotensin system is involved.

## Author contributions

The study was designed by MR-S and performed by AS, IP, and MR-S. The manuscript was written by MR-S and revised by MdG and MM-C. Statistical analysis was performed by JL. All authors provided feedback on manuscript.

### Conflict of interest statement

The authors declare that the research was conducted in the absence of any commercial or financial relationships that could be construed as a potential conflict of interest.
